# Toll-like Receptor 7 Controls the Anti-Retroviral Germinal Center Response

**DOI:** 10.1371/journal.ppat.1002293

**Published:** 2011-10-06

**Authors:** Edward P. Browne

**Affiliations:** Koch Institute for Integrative Cancer Research, Massachusetts Institute of Technology, Cambridge, Massachusetts, United States of America; University of Pennsylvania School of Medicine, United States of America

## Abstract

The development of vaccines that can enhance immunity to viral pathogens is an important goal. However, the innate molecular pathways that regulate the strength and quality of the immune response remain largely uncharacterized. To define the role of Toll-like receptor (TLR) signaling in control of a model retroviral pathogen, Friend virus (FV), I generated mice in which the TLR signaling adapter Myd88 was selectively deleted in dendritic cell (DC) or in B cell lineages. Deletion of Myd88 in DCs had little effect on immune control of FV, while B cell specific deletion of Myd88 caused a dramatic increase in viral infectious centers and a significantly reduced antibody response, indicating that B cell-intrinsic TLR signaling plays a crucial role, while TLR signaling in DCs is less important. I then identified the single-stranded RNA sensing protein TLR7 as being required for antibody-mediated control of FV by analyzing mice deficient in TLR7. Remarkably, B cells in infected TLR7-deficient mice upregulated CD69 and CD86 early in infection, but failed to develop into germinal center B cells. CD4 T cell responses were also attenuated in the absence of TLR7, but CD8 responses were TLR7 independent, suggesting the existence of additional pathways for detection of retroviral particles. Together these results demonstrate that the vertebrate immune system detects retroviruses *in vivo* via TLR7 and that this pathway regulates a key checkpoint controlling development of germinal center B cells.

## Author Summary

Viral infection triggers potent pathogen-specific immune responses involving antibodies that neutralize viral particles and CD8 T cells that directly kill infected cells. Vaccines also trigger immune responses, but current vaccines for retroviruses such as HIV-1, are inadequate. Defining the genes and pathways that regulate this response will identify new targets for therapies that can enhance the immune response to infection or to prophylactic vaccines. Using mouse genetics, I have demonstrated that a host protein, Toll-like receptor seven (TLR7) recognizes retroviruses and regulates the antibody response to infection. TLR7 is a member of an ancient family of genes that detect microbes and initiate inflammation, but its role in antibody responses has not been clearly defined. I have discovered that TLR7 controls a specific step in the antibody response called the germinal center reaction. Germinal centers regulate the development of antibodies that protect against viral infection, and manipulation of TLR7 and its signaling pathway in B cells could be a viable strategy for enhancing immunity to viruses.

## Introduction

The retrovirus family includes several human pathogens, such as HIV-1, HIV-2 and HTLV-1, for which no effective vaccine exists [1], [2], [3]. Efforts to induce broadly neutralizing antibodies against HIV-1 by vaccination with monomeric gp120 have produced disappointing results for reasons that are not entirely clear [4]. The high mutation rate of the envelope glycoprotein, and glycosylation of neutralizing epitopes, are likely contributing factors [5], [6]. Early during natural HIV-1 infection, abundant antibodies to gp120 are produced but these fail to neutralize the virus. Some individuals eventually produce broadly neutralizing antibodies, but these typically arise too late to be of clinical benefit [7]. Vaccines for other viruses, such as influenza, face similar issues of viral diversity and mutation. Thus, identifying ways to improve the speed and quality of the antibody response to infection and vaccination is a key priority. Specifically, it will be crucial to identify host genetic pathways that contribute to the development of anti-viral neutralizing antibodies and to develop strategies that target these pathways.

Over the past decade it has become clear that the innate immune system is an important contributor to the activation and fine-tuning of adaptive immune responses, but the precise details of how these pathways contribute are still unclear for most pathogens [8]. In particular, the identity of proteins that ‘sense’ the presence of viral particles and the details of how they shape adaptive immunity will need to be elucidated [9]. Innate sensors of microbial infection fall into three basic classes, NOD like receptors (NLRs), RIG-I like receptors (RLRs) and Toll-like receptors (TLRs). Each family consists of several members that have specialized functions. Mice deficient in individual pathogen-sensing proteins have been constructed in several laboratories and have been analyzed for effects on innate and adaptive immunity to viral pathogens [10].

Our knowledge of how innate sensing pathways regulate adaptive immunity to HIV-1 has been hampered by the lack of a genetically modifiable animal model for HIV-1 infection. HIV-1 infection of murine cells is blocked at multiple steps in the viral replication cycle [11]. Friend virus (FV) is a murine gammaretrovirus that has been widely used as a model to understand basic principles of retroviral immunology [12]. FV consists of a replication-competent virus (F-MLV) and a defective spleen focus-forming virus (SFFV). Infection of C57BL/6 mice with FV induces a potent CD8 T cell and antibody response that controls the initial infection, although the mice eventually develop a low-level persistent infection [13]. This system thus allows the application of the powerful tools of mouse genetics to the question of how innate immune pathways regulate adaptive immunity to retroviral pathogens.

It was recently shown that mice deficient in the TLR adapter Myd88 exhibit a profound deficit in immune control of FV, and mount an attenuated antibody response to the virus, indicating that a member of the TLR family is involved in antibody-mediated immune control [14]. However, the identity of the specific TLR that detects FV, and the cell lineages where its signaling is required, are unknown. I sought to identity the cell lineage specific requirements for Myd88 expression in the anti-FV antibody response, and to identify individual TLR family members that contribute to retroviral immunity. The data revealed that B cell-intrinsic Myd88 and TLR7 play a key role in the antibody response to infection, and that they regulate the development of germinal center B cells. Since germinal center reactions are a key process that controls the quality of the antibody response, these finding have broad significance for understanding the immune response to viruses with RNA genomes, and the design of vaccine vectors.

## Methods and Materials

### Ethics statement

This study was carried out in strict accordance with the recommendations in the Guide for the Care and Use of Laboratory Animals of the National Institutes of Health. These studies were approved by the Committee on Animal Care of the Massachusetts Institute of Technology (protocol #0709-088-12).

### Mice

Myd88 knockout mice were originally derived in the laboratory of Shizuo Akira (Osaka). All other mice were purchased from The Jackson Laboratory (Bar Harbor). The specific strain/stock numbers for the mice were: C57BL/6 (000664), BALB/c (00651), TLR7-deficient mice: B6.129S1-Tlr7tm1Flv/J (008380), Floxed Myd88 allele mice: B6.129P2(SJL)-Myd88tm1Defr/J (008888), CD19-cre: B6.129P2(C)-Cd19tm1(cre)Cgn/J (006785), and CD11c-cre: C57BL/6J-Tg(Itgax-cre,-EGFP)4097Ach/J (007567). All knockout and transgenic strains had been backcrossed to a C57BL/6 background for multiple generations.

### Virus stocks and infections

All infections were carried out on mice at 6–8 weeks of age. A stock of Lactate dehydrogenase-elevating virus (LDV) free FV was obtained from Kim Hasenkrug (NIAID). Stocks of FV were prepared by retro-orbital infection of BALB/c mice and harvesting spleens at 8 dpi. 10% spleen homogenates were prepared and stored at −80°C. The virus stock was confirmed to be free of LDV and other pathogens by PCR screening.

Virus stocks and experimental samples were quantified by focus-forming assay. Serial dilutions of splenocytes were plated on *Mus dunni* cells in RPMI media. Two days later, cells were fixed in ethanol and stained with the FV envelope-specific monoclonal antibody mAb720 [15] (generous gift from Kim Hasenkrug, NIAID) then with anti-IgG1 horseradish peroxidase (BD Pharmingen). Foci were developed with aminoethyl-carbazole substrate in a 0.1 M sodium citrate buffer with hydrogen peroxide. For experimental infections, mice were dosed with 10000 focus-forming units of FV by retro-orbital injection.

### Antibody assays

To determine FV envelope-specific antibody levels in mice, I obtained serum by eyebleed. To measure total anti-FV Ig levels, I used serum diluted 1∶10 in phosphate buffered saline (PBS) to stain a chronically F-MLV and SFFV-infected rat kidney cell line that abundantly expresses the FV envelope protein (Generous gift of Leonard Evans, NIAID). These cells were then washed and stained with an allophycocyanin conjugated polyclonal anti-IgG(H+L) antibody (ebiosciences) and analyzed by flow cytometry on an Accuri C6 flow cytometer. To measure neutralizing antibody levels, I performed a standard neutralization assay: 50 focus-forming units of virus was incubated with serum from infected mice over a range of dilutions at room temperature for 30 mins. The samples were then plated on *Mus dunni* cells, and the number of foci counted at 2 dpi. The neutralizing potency of the serum was defined as the maximum serum dilution capable of reducing the number of foci by 50% or greater.

### Cell staining and flow cytometry

For staining of mouse splenocytes, a single-cell suspension was generated and erythrocytes removed by hypotonic lysis. Cells were stained in PBS with 2% FBS at 4°C for 30 minutes, then washed with PBS and fixed in 2% paraformaldehyde for 20 minutes. Samples were analyzed on an Accuri C6 flow cytometer. For intracellular IFNγ staining, cells were first restimulated for 3 h in PMA/ionomycin, permeabilized and fixed in Perm/Fix buffer (BD Pharmingen), then washed in Perm/Wash buffer and stained with anti-IFNγ-FITC. Other antibodies used were: αCD19-FITC, αCD19-PercpCy5.5, αCD69-FITC, αPD1-PE, αGL7-Alexa-647, αTCRβ-APC, αCD4-PercPCy5.5, αCD8-PE, and αIgM-PE (all from ebiosciences). H2D^b^-GagL-PE tetramer was obtained from Beckman-Coulter. All stainings were performed in the presence of an Fc blocking antibody (ebiosciences).

### Histology

Mouse spleens were fixed in 4% paraformaldehyde overnight, then embedded in paraffin. 4 µm Sections were cut and mounted on glass slides, then stained with hematoxylin and alcoholic eosin solutions for 1 minute each. The stained sections were then overlaid with a 90% glycerol solution and coverslips before microscopic analysis.

### Statistical analyses

P values for experiments were determined by Student's t test, except for viral titers, which were analyzed by the Mann-Whitney method.

### Accession numbers

**TLR7**: CAM14953

**Myd88**: AAC53013

## Results

### B cell-intrinsic TLR signaling is required for control of FV

FV infection leads to an acute viremia that peaks at 7–8 days post infection, but is brought under control by potent CD8 T cell and B cell responses by 14 dpi in resistant strains of mice [12]. The TLR adapter Myd88 is required for the generation of a potent serum immunoglobulin (Ig) response to the virus [14]. Since Myd88 is widely expressed in the vertebrate immune system and also in non-immune lineages [16], the requirement for Myd88 in the antibody response to FV could reflect its activity in any or several of these cell types. I examined the role of TLR signaling in two immune lineages known to contribute to anti-FV antibody responses - dendritic cells (DCs) and B cells. Dendritic cells (DCs) are required for the development of FV-specific antibodies [14], and express a number of different TLRs. Murine B cells also highly express several TLRs, including TLR4, TLR7, and TLR9. To define the cell-intrinsic requirements for Myd88 further, I crossed mice containing a ‘floxed’ allele of *Myd88* with strains that express the Cre recombinase selectively in dendritic cells (CD11c-Cre) or in B cells (CD19-Cre). The ‘floxed’ *Myd88* allele mice have been previously used to demonstrate the DC-intrinsic requirement for TLR signaling in control of *Salmonella* [17].

**Figure 1:**
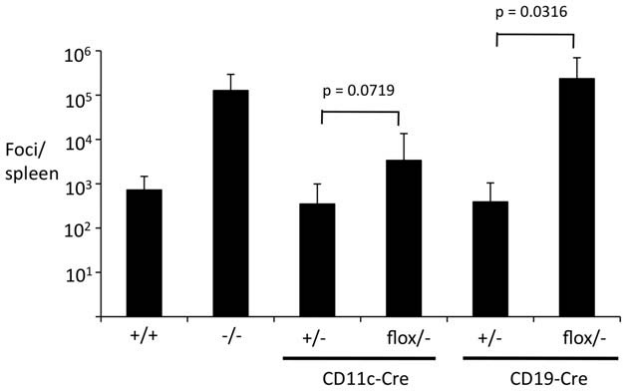
B cell-intrinsic TLR signaling is required for control of FV. Mice that were heterozygous (+/−) or knockout (−/−) for Myd88, and mice with Myd88 deleted in DCs (CD11c-Cre/*Myd88^flox/−^*) or B cells (CD19-Cre/*Myd88^flox/−^*) were infected with FV, and viral infectious centers were determined at 14dpi by measuring focus-forming units per spleen. Each bar represents an average of 6–10 mice.

To determine whether DC or B cell-intrinsic Myd88 expression is required for control of FV infection, I infected the conditionally deleted mouse strains with FV. Consistent with previous findings [14], germline Myd88-deficient mice exhibited dramatically elevated numbers of viral infectious centers at 14 dpi (Fig. 1). Surprisingly, mice with Myd88 selectively deleted in DCs (CD11c-Cre/*Myd88^flox/−^*) showed only a small increase in infectious centers at 14 dpi, suggesting that DC-intrinsic TLR signaling makes only a minor contribution to control of FV. By contrast, mice with Myd88 deleted in B cells (CD19-Cre/*Myd88^flox/−^*) exhibited a profound defect in control of FV, and had dramatically higher numbers of infectious centers than non-deleted mice (Fig. 1). These results indicate that B cell-intrinsic TLR signaling plays an essential role in the control of FV, while DC-intrinsic TLR signaling is less important.

### B cell-intrinsic Myd88 is required for an antibody response to FV

**Figure 2:**
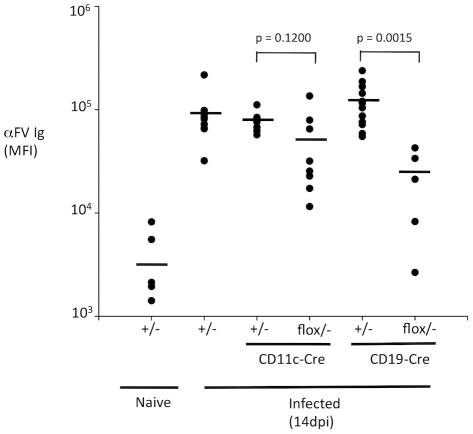
B cell-intrinsic TLR signaling is required for an antibody response to FV. Myd88 germline heterozygous mice (+/−) and mice with Myd88 deleted in DCs (CD11c-Cre/*Myd88^flox/−^*) or B cells (CD19-Cre/*Myd88^flox/−^*) were infected with FV, and FV-specific Ig levels in the serum determined at 14 dpi by staining of envelope expressing cells and flow cytometry. Units represent the mean fluorescence intensity (MFI) of cells stained with diluted serum, followed by allophycocyanin conjugated. anti-mouse IgG(H+L). Each dot represents an individual mouse.

The high levels of viral foci observed in mice with B cell specific deletion of Myd88 could reflect a requirement for B cell-intrinsic Myd88 in the antibody response, or it could reflect an antibody-independent role for B cell-intrinsic Myd88 in control of FV. To determine whether conditional deletion of Myd88 in DCs or B cells affected the antibody response to FV, I measured total FV-specific Ig levels in the serum of infected mice with Myd88 deleted in DCs or in B cells at 14 dpi. Mice with Myd88 deleted in DCs showed a small reduction in antibody titers at 14 dpi compared to non-deleted littermate^−^ mice (Fig. 2), but this reduction was not statistically significant. By contrast, mice with Myd88 deleted in B cells showed a strong reduction in antibody levels compared to undeleted littermate mice. These data demonstrate that B cell-intrinsic Myd88 plays a direct role in the regulation of the antibody response to FV.

### TLR7 is required for an antibody response to FV

**Figure 3:**
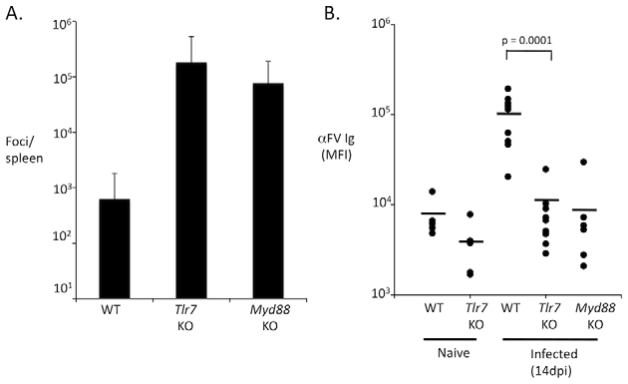
TLR7 is required for an antibody response to FV. **A.** Wild-type (WT) or TLR7 deficient mice (*Tlr7* KO) were infected with FV. At 14 dpi, spleens from infected mice were harvested and the number of infectious focus-forming units per spleen was measured by focus-forming assay. Each bar represents the average of 6 mice. **B.** The levels of FV-specific Ig in the serum of infected and uninfected mice were measured by staining of an envelope expressing cell line and flow cytometry. Units represent the mean fluorescence intensity (MFI) of cells stained with diluted serum, followed by allophycocyanin conjugated. anti-mouse IgG(H+L). Each dot represents an individual mouse. The average is shown as a horizontal black bar.

Myd88 mediates signaling from all members of the TLR family except TLR3, as well as signaling from IL1R and IL18R [18], [19]. Each member of this family has a specialized function relating to the recognition of conserved molecular patterns found on microbes. TLR7 and TLR9 have been shown to respond to single stranded RNA (ssRNA) and double stranded DNA (dsDNA) elements respectively, while TLR4 responds to lipopolysaccharide and oxidized phospholipids [20], [21], [22]. Since retroviruses have ssRNA genomes that could stimulate TLR7 [23], and TLR7 is abundantly expressed in B cells, I hypothesized that TLR7 regulates the antibody response to FV. To test this hypothesis, I infected wild-type and TLR7-deficient mice with FV and measured viral infectious centers at 14 dpi. Wild-type mice exhibited low numbers of infectious centers at 14 dpi, consistent with previous observations (Fig. 3A). By contrast, TLR7-deficient mice exhibited dramatically higher levels of infectious centers, indicating a significant defect in immune control of FV. This demonstrates that TLR7 is indeed a key host factor that regulates control of retroviral infection *in vivo*.

To determine whether TLR7 regulates the antibody response to FV, I measured total FV-specific Ig levels in the serum of infected wild-type or TLR7-deficient mice at 14 dpi. As expected, wild-type mice exhibited a robust serum FV-specific Ig response to FV at 14 dpi (Fig. 3B). Notably, TLR7-deficient mice exhibited a profoundly attenuated antibody response to FV. Myd88 and TLR7 deficient mice exhibited similarly low levels of FV-specific Ig, indicating that a lack of TLR7 signaling likely accounts for the defect observed in Myd88 knockout mice. Together, these results identify TLR7 as a key regulator of the antibody response to retroviral infection.

**Figure 4:**
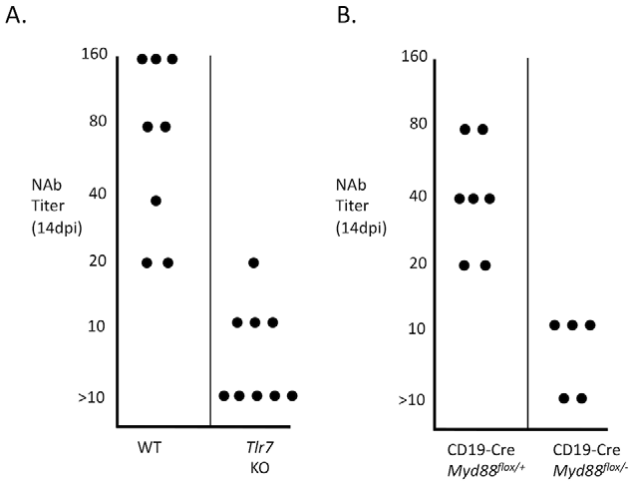
TLR7 and B cell-intrinsic Myd88 are required for a neutralizing antibody response. Serum from infected wild-type (WT) or TLR7-deficient mice (*Tlr7* KO) (**A**), as well as mice with Myd88 deleted in B cells (CD19-Cre/Myd88^flox/−^) (**B**), was diluted over a wide range and incubated with a defined quantity of infectious FV. The effect of the serum on the infectivity of the sample was then measured by focus-forming assay. The neutralizing antibody (NAb) titer was defined as the maximum serum dilution that reduced the infectivity of the virus sample by at least 50%. Each dot represents an individual mouse.

I also confirmed the effect of B cell-intrinsic Myd88 and TLR7 on the antibody response to FV by performing neutralization assays using serum from infected wild-type mice, TLR7-deficient mice, or mice with Myd88 deleted in B cells (Fig. 4). Serum from wild-type mice was able to potently neutralize FV samples *in vitro*. By contrast, serum from TLR7-deficient mice or mice with Myd88 deleted in B cells exhibited significantly attenuated neutralizing ability.

### B cells upregulate CD86 and CD69 early during infection independently of TLR7

**Figure 5:**
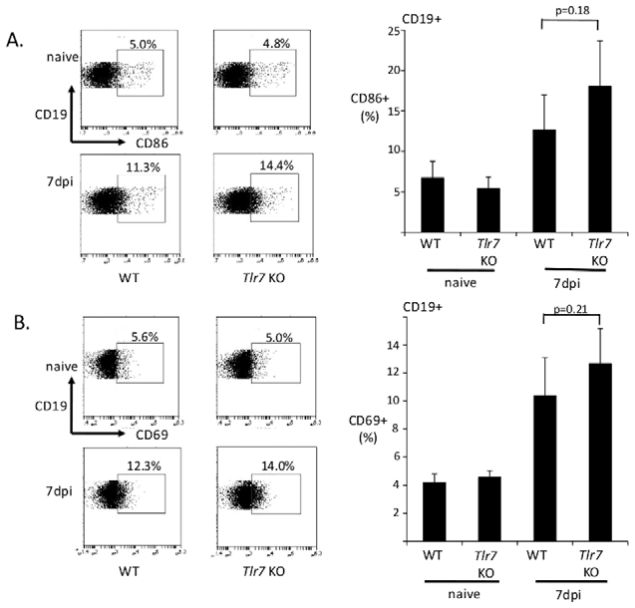
B cells upregulate CD86 and CD69 independently of TLR7. Wild-type (WT) or TLR7-deficient mice were infected with FV. At 7 dpi, splenocytes were harvested and the levels of CD86 (**A**) and CD69 (**B**) expression determined for B cells (CD19+) by flow cytometry. A representative flow cytometry plot for the CD19+ gated cells for each group is shown. Each bar of the histogram represents the average of 5–6 mice. Control ‘naïve’ mice were inoculated with an equivalent dose of spleen homogenate without FV.

Antibody responses to viral infection are regulated by a network of cells and signaling pathways, including dendritic cells (DCs), CD4 T cells and B cells. Naïve B cells interact with viral antigen through the B cell receptor (BCR), and with CD4 T cells through a number of cell surface molecules, including CD40. Activation initially results in the upregulation of markers such as CD69 and CD86. Activated B cells can then develop into germinal center (GC) B cells with the assistance of CD4 T cells [24]. To determine whether early activation of B cells is regulated by TLR7, I examined expression of CD69 and CD86 on B cells at 7 dpi. To rule out potential FV independent effects from the spleen homogenate used for infection, I compared infected mice to mice dosed with an equivalent amount of spleen homogenate from an uninfected mouse. B cells in infected mice upregulated expression of CD69 and CD86 at 7 dpi in wild-type infected mice, indicating that B cell activation is occurring (Fig. 5). Interestingly, this upregulation also occurred in TLR7-deficient mice. This suggests that *in vivo* activation of B cells does not require TLR7, and indicates that this early activation is triggered by a TLR7 independent signaling pathway.

### TLR7 regulates the formation of germinal center B cells during FV infection

Since early activation of B cells was apparently normal in infected TLR7-deficient mice, I hypothesized that TLR7 regulates a later step in the antibody response, such as the development of germinal center B cells. Germinal center B cells upregulate expression of GL7 and undergo Activation Induced Deaminase (AID) dependent somatic hypermutation and switching to non-IgM isotypes [25], [26], [27]. This process thus contributes the development of affinity-matured and class switched neutralizing antibodies [28]. To address the role of TLR7 in GC reactions, I analyzed the development of germinal center B cells during FV infection in wild-type and TLR7-deficient mice by flow cytometry.

**Figure 6:**
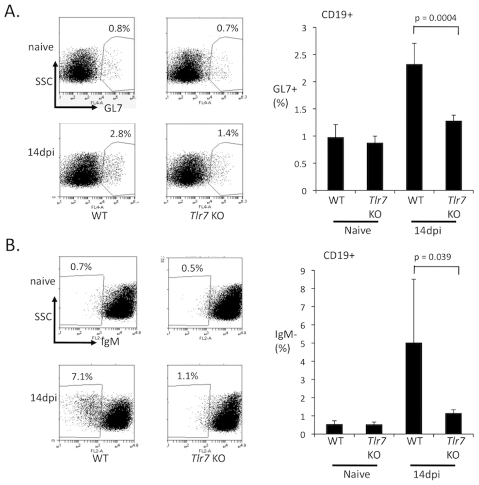
TLR7 is required for the development of germinal center B cells during FV infection. Wild type (WT) or TLR7-deficient mice (*Tlr7* KO) were infected with FV. At 14 dpi, splenocytes were harvested and analyzed for the presence of germinal center B cells by flow cytometry. The percentage of B cells that were (**A**) CD19+, GL7+ or (**B**) CD19+, IgM− were measured and plotted. A representative plot for the CD19+ gated cells for each group is shown. Each bar of the histogram represents the average of 5–6 mice.

**Figure 7:**
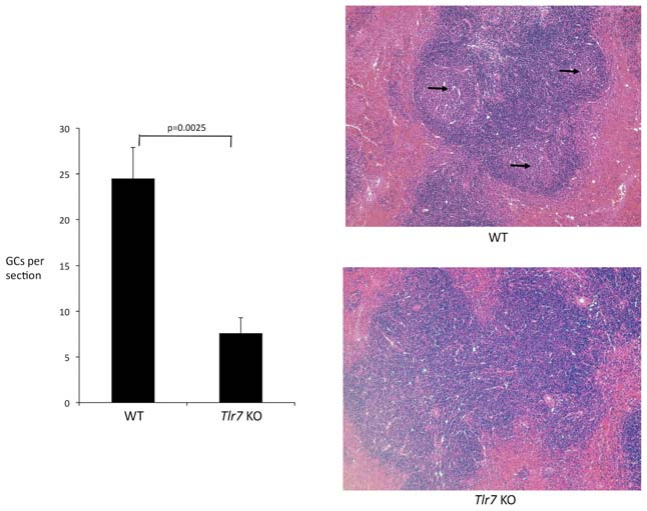
Histological analysis of germinal center formation during FV infection. Spleens were harvested from FV-infected wild-type (WT) or TLR7 deficient (*Tlr7* KO) mice at 14 dpi, embedded in paraffin, and sectioned onto glass slides. Sections were stained with hematoxylin and eosin. Germinal centers (GCs) were identified by their characteristic staining pattern of a paler central circular area (indicated by arrows) surrounded by a darker mantle zone and marginal zone. Five mice for each group were analyzed, and four non-consecutive sections were analyzed for each mouse.

At 14 dpi infection B cells in wild-type mice had upregulated the germinal center marker GL7, and had developed a significant population of non-IgM B cells, indicating that class switching and germinal center reactions were occurring (Fig. 6). In TLR7-deficient mice, by contrast, the level of GL7+ and non-IgM B cells were significantly lower. I also examined the germinal centers in the spleens of infected mice by histology. Wild-type infected mice exhibited abundant germinal center structures by 14 dpi, while in the TLR7-deficient mice, germinal centers were significantly reduced (Fig. 7). These results suggest that TLR7 signaling regulates a post-activation checkpoint that controls the formation or maintenance of germinal center B reactions during FV infection.

### TLR7 regulates IFNγ expression in the CD4 but not the CD8 T cell response

**Figure 8:**
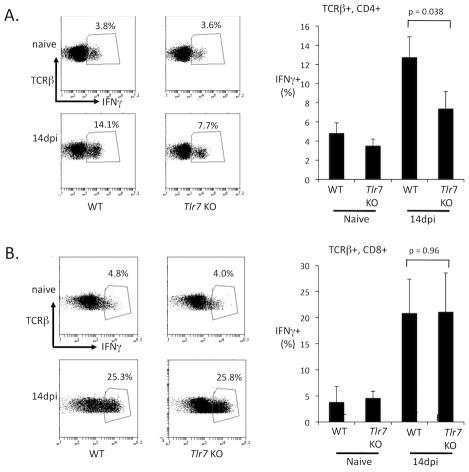
TLR7 is required for IFNγ expression in CD4 T cells. Wild-type (WT) and TLR7 deficient (*Tlr7* KO) mice were infected with FV. At 14 dpi, splenocytes were harvested, restimulated for 3 h with PMA/ionomoycin, and analyzed by flow cytometry for intracellular expression of IFNγ in (**A**) CD4 T cells (TCRβ+, CD4+) and (**B**) CD8 T cells (TCRβ+, CD8+). A representative flow cytometry plot for the T cell gated cells for each group is shown. Each bar of the histogram represents the average of 5–6 mice.

**Figure 9:**
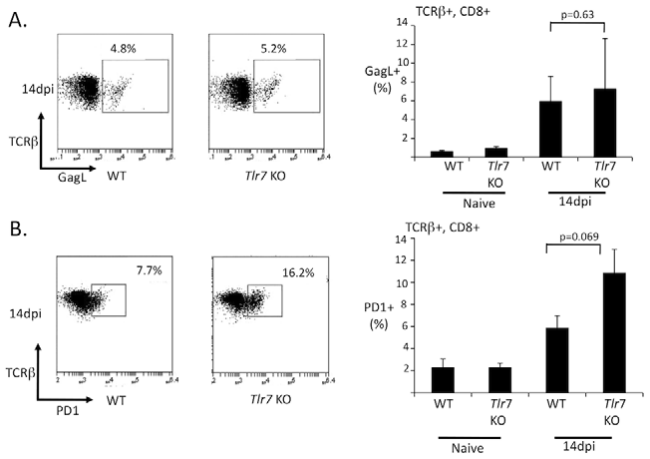
The FV-specific CD8 T cell response is independent of TLR7. Wild-type (WT) or TLR7 deficient mice (*Tlr7* KO) were infected with FV. At 14 dpi, splenocyte suspensions were analyzed by flow cytometry with an H2D^b^-GagL tetramer (**A**), or with an antibody to detect PD1 expression (**B**). Flow cytometry plots are gated on TCRβ+, CD8+ T cells. Each bar of the histogram represents the average of 5–6 mice.

Since CD4 T cells are important regulators of germinal center responses, the attenuated germinal response to FV in TLR7-deficient mice could reflect defective CD4 T cell help. To examine whether TLR7 was required for T cell responses during FV infection, wild-type or TLR7-deficient mice were infected with FV, and at 14 dpi, the expression of IFNγ in CD4 and CD8 T cells was measured by intracellular cytokine staining. Wild-type infected mice strongly upregulated IFNγ expression in CD4 and CD8 T cells, indicating a robust T cell response to FV. Interestingly, IFNγ upregulation in CD4 T cells was dependent on TLR7 (Fig. 8A), while IFNγ expression in CD8 T cells was TLR7 independent (Fig. 8B). I also examined the FV-specific CD8 T cell response using an H2D^b^ tetramer complexed with the FV GagL peptide (Fig. 9A). I found that the development of GagL specific CD8 T cells at 14 dpi was independent of TLR7. Since TLR7 mice have significantly higher viral loads at 14 dpi than wild-type mice, I examined whether the CD8 T cells of TLR7-deficient mice exhibited higher levels of antigen-driven exhaustion by staining for PD1 expression (Fig. 9B). Although I observed a higher number of PD1+ CD8 T cells in TLR7 deficient mice compared to wild-type mice, this difference was not statistically significant. These data suggest that TLR7 selectively affects pathways that regulate CD4 T cell responses, while TLR7-independent pathways are sufficient for CD8 T cell activation. Defective CD4 T cell function in TLR7-deficient mice could contribute to the inadequate antibody response and the reduced germinal center response.

### B cell-intrinsic Myd88 regulates germinal center B cells but not IFNγ expression in CD4 T cells

**Figure 10:**
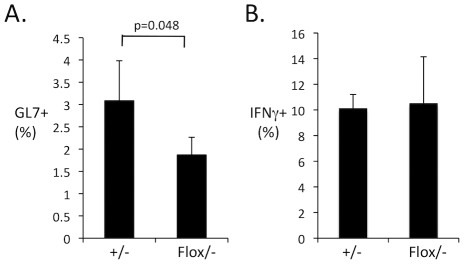
B cell-intrinsic Myd88 regulates germinal center B cells but not CD4 T cell IFNγ expression. Heterozygous mice (CD19-Cre/Myd88^+/−^) or mice with B cell specific deletion of Myd88 (CD19-Cre/Myd88^flox/−^) were infected with FV, At 14 dpi, splenocytes suspensions were analyzed by flow cytometry for GL7 expression in CD19+ B cells (**A**) and for IFNγ expression in CD4+, TCRβ+ T cells (**B**). Each bar represents the average of four mice.

Since, B cell-intrinsic Myd88 is important for the antibody response I wished to determine whether B cell-intrinsic Myd88 was required for either germinal center responses or CD4 T cell IFNγ expression. I examined GL7 expression in splenic B cells during FV infection of heterozygous mice or mice with Myd88 selectively deleted in B cells at 14 dpi (Fig. 10A). I also examined CD4 T cell IFNγ expression in these mice by intracellular cytokine staining at 14 dpi (Fig. 10B). Interestingly, I found that GL7 expression was significantly reduced in infected mice with B cell deleted Myd88, while IFNγ expression was unaffected. This demonstrates that B cell-intrinsic Myd88 is required for germinal center responses but not for IFNγ expression in CD4 T cells. Thus it is possible that TLR7 signaling in a cell lineage other than B cells regulates CD4 T cell expression of IFNγ.

## Discussion

In this study I present evidence that B cell-intrinsic Myd88 expression and TLR7 are key regulators of the germinal center response to a retroviral pathogen, FV. TLR7-deficient mice fail to develop a potent serum antibody response after infection with FV, and this correlates with a significant reduction in the formation of GL7+ germinal center B cells. Since TLR7 is abundantly expressed in B cells, it is likely that these results reflect a B cell-intrinsic requirement for TLR7, although I cannot yet rule out the possibility that other cell lineages contribute to TLR7-mediated control of FV, or that other TLRs contribute to B cell-intrinsic recognition of FV. Indeed, since IFNγ expression in CD4 T cells was defective in TLR7 deficient mice but not in mice lacking B cell-intrinsic Myd88, it is likely that this response is regulated in part by B cell extrinsic TLR7 signaling.

The issue of whether TLRs contribute to B cell responses has been controversial. Pasare and coworkers reported that B cell-deficient mice reconstituted with B cells from Myd88 knockout mice had reduced antibody responses to OVA with LPS [29]. This finding was challenged by other groups that found apparently normal antibody responses to trinitrophenol-hemocyanin with Complete Freund's Adjuvant in mice with defective TLR signaling [30], [31]. The results presented in this study strongly support the hypothesis that B cell-intrinsic TLR signaling can regulate antibody responses, and also suggest that this process specifically regulates the development of germinal centers during viral infection. I also found that DC-intrinsic TLR signaling makes only a minor contribution to the anti-retroviral antibody response. Previous work has shown that deletion of CD11c+ DCs with diphtheria toxin causes a dramatic attenuation of the antibody response to FV [14], demonstrating that CD11c+ DCs make an essential contribution of the antibody response. I therefore speculate that a TLR-independent innate pathway contributes to the role of DCs in the immune response to FV. This notion is supported by my observation that the CD8 T cell response to FV, which is regulated by CD11c+ dendritic cells, is TLR7 independent. The TLR7 independent innate signaling pathway that regulates to the CD8 T cell IFNγ response is unknown.

Recent work by other laboratories examining the role of TLR signaling in the antibody response to influenza and model antigens have also indicated that B cell-intrinsic TLR activation controls germinal center responses [32], [33], [34], [35]. Also, B6.Yaa mice, which contain a duplicated *Tlr7* gene, display enhanced germinal center and antibody responses to immunization [36]. Thus, a requirement for B cell-intrinsic TLR7 signaling may be a general feature of the antibody response to viruses with RNA genomes. Targeted delivery of TLR agonists to B cells with synthetic nano-particles may enhance germinal center responses [33].

Determining how TLR7 regulates the development of germinal center B cells during viral infection should be an area of further investigation. TLR7 stimulation by viral RNA may directly regulate the expression of a set of transcription factors or genes that promote the initiation of germinal center reactions and immunoglobulin class switching, such as Activation Induced Deaminase (AID) [37]. Alternatively, it may regulate expression of cytokines that mediate autocrine effects on B cells [38]. TLR7 may also regulate the maintenance of GC responses by stimulating ongoing proliferation or survival of GC B cells.

Germinal center B cells are known to be regulated by a specialized population of CD4+ T cells called “follicular helper” cells (Tfh) [24]. Tfh cells are in turn negatively regulated by CD44+, CD122+ CD8+ regulatory cells, by a mechanism that requires IL15 expression [36], [39]. It is possible that TLR7 signaling acts by either promoting Tfh proliferation or function, or by interfering with CD8 Treg repression of Tfh cells. Previous data have suggested that CD4+ CD25+ regulatory T cells are deactivated by TLR stimulation of dendritic cells, via a mechanism involving IL6 [40]. As such, a similar regulatory model could apply to inhibition of antibody responses by CD8+ regulatory T cells; TLR7 activation by viral RNA may promote antibody responses via a secondary effect on CD8+ Tregs.

Our data also suggest the existence of a novel uncharacterized retrovirus sensing pathway that controls CD8 T cell responses to infection. The identity of this sensing pathway is unknown, and its identification could have significant implications for understanding CD8 T cell responses to human retroviral pathogens such as HIV-1. It could involve reverse transcribed retroviral DNA being sensed via DNA sensing proteins such as ZBP1 [41], or viral RNA being detected by RIG-I [42]. Although RIG-I detects 5′ triphosphates, and the 5′ ends of retroviral RNAs are typically modified by a 5′ methyl-guanosine cap, it has recently been shown that cytosolic dsDNA can serve as a template for the generation of short RNAs that can trigger RIG-I [43], [44]. It has also recently been shown that HIV-1 Gag expression can, in the presence of vpx from SIV, promote maturation of human monocyte derived dendritic cells, although it is unclear if this pathway contributes to natural immunity to HIV-1 [45]. FV infected cells were also recently shown to express the NKG2D ligand Rae-1 [46]. The cytidine deaminase Apobec3 has also been shown to modulate B cell responses to FV [47], although this is most likely through an indirect mechanism [48], [49].

The finding that TLR7 and B cell-intrinsic Myd88 regulate the antibody response to a retroviral pathogen by promoting the development of germinal center B cells has potentially significant implications for understanding mechanisms behind the antibody response to human retroviral pathogens such as HIV-1 and HTLV-1. The initial antibody response to HIV-1 is rapid but not neutralizing, followed by autologous neutralizing antibody response and viral escape. In a minority of patients, broadly neutralizing antibodies eventually develop but often not until 2–3 years post infection [7]. Interestingly, the development of broadly neutralizing antibodies has been shown to correlate with higher viral titers and with higher expression of the Tfh cell marker PD1 in CD4 T cells [7]. HIV-1 envelope protein has been shown to disrupt TLR7 activation in DCs by inhibiting the formation of autophagic vesicles that deliver viral TLR7 ligands to endosomes [50]. It is thus possible that HIV-1 suppression of TLR7 activation could affect the antigen-specific antibody response to HIV-1. Conversely, non-specific stimulation of B cells via TLR7 could contribute to polyclonal B cell activation and exhaustion during HIV-1 infection [51]. A key area of investigation for future studies will be to identify host factors that contribute specifically to the breadth on the anti-envelope antibody response as opposed to total level of antibodies.

### Additional note

While this manuscript was in revision, another group reported findings that confirm the role of TLR7 in the antibody response to murine retroviral infection [52].
